# Supplementary material for: Exploring the Contribution of TLR7 to Sex-Based Disparities in Respiratory Syncytial Virus (RSV)-Induced Inflammation and Immunity
Source: Viruses. 2025 Mar 16;17(3):428. doi: 10.3390/v17030428 (PMC11946665; doi:10.3390/v17030428)
Supplement: Supplementary file 1 [file viruses-17-00428-s001.zip › viruses-3516635-supplementary.pdf]

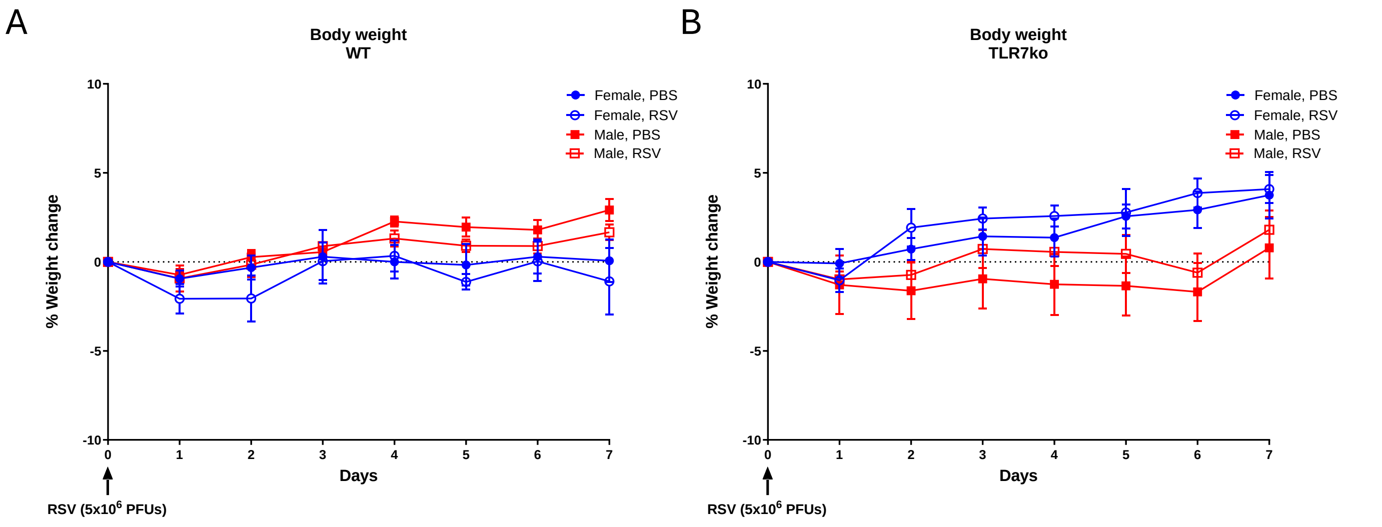

Figure S1. RSV infection does not cause body weight loss in male or female mice. Male or female (A) WT C57Bl/6 or (B) TLR7ko mice were infected with RSV-A Long ($5x10^6$ PFUs) or mock-infected with PBS. Bodyweights were recorded daily for 7 days and presented as % weight change from day of infection. Data is expressed as mean ± SEM, n = 5-7. Statistical analysis was conducted using two-way ANOVA test followed by Tukey's post hoc test for multiple comparisons ($p > 0.05$).

A

Gene (Lungs)	Sex difference (WT)	Sex difference (TLR7ko)
IL1B	↓♂	No response to infection
NLRP3	↓♂	↓♂
IL18	↓♂	↓♂
IL6	↓♂	No response to infection
TNFA	↑♂	No difference
IL4	↓♂	No response to infection
IL5	↑♂	↑♂
IL13	↓♂	No response to infection
IFNG	↓♂	↓♂
IFNB	↓♂	No response to infection
IFNL3	No difference	No difference
IRF7	↓♂	No difference
CXCL2	↓♂	No response to infection
CCL2	↓♂	↓♂
CCL3	↓♂	↓♂
CCL5	↓♂	↓♂
RSV F	↓♂	↓♂

B

Cell type (BALF)	Sex difference (WT)	Sex difference (TLR7ko)
Total live cells	No difference	No difference
Macrophages	↓♂	No difference
Neutrophils	No difference	↓♂
Lymphocytes	No difference	↑♂
Eosinophils	No difference	No difference

C

Isotype (BALF)	Sex difference (WT)	Sex difference (TLR7ko)
IgA	No difference	No response to infection
IgE	↓♂	↑♂
IgG1	No difference	No difference
IgG2a	↓♂	No response to infection
IgG2b	↓♂	No difference
IgG3	↓♂	↑♂
IgM	↓♂	No difference

Figure S2. Summary of sex differences following RSV infection. Comparison of (A) lung gene expression, (B) immune cell types in the BALF or (C) antibody isotypes in the BALF between male and female mice of WT or TLR7ko genotype.
